# De Novo Missense Variant in Bovine *WDR33* Associated With a Complex Syndromic Form of Cleft Palate With Pentalogy of Fallot and Internal Hydrocephalus

**DOI:** 10.1111/jvim.70144

**Published:** 2025-06-17

**Authors:** Marilena Bolcato, Giovanni Romito, Irene M. Häfliger, Arcangelo Gentile, Cord Drögemüller, Joana G. P. Jacinto

**Affiliations:** ^1^ Department of Veterinary Medical Sciences University of Bologna Ozzano Italy; ^2^ Institute of Genetics, Vetsuisse Faculty University of Bern Bern Switzerland; ^3^ Clinic for Ruminants, Vetsuisse Faculty University of Bern Bern Switzerland

**Keywords:** cattle, CNS disorders, congenital heart defects, germinal mosaicism, palatoschisis, pentalogy of Fallot, precision medicine

## Abstract

**Background:**

Cleft palate (CP) is a congenital defect characterized by an opening in the palate. Two crossbred paternal half‐sibs with a complex syndrome including CP were identified.

**Hypothesis/Objectives:**

Characterize disease phenotype and evaluate the genetic cause of the observed syndrome.

**Animals:**

Two affected calves, their parents, and 5576 control cattle genomes.

**Methods:**

The affected animals were evaluated clinicopathologically. Paternal half‐sibling trio‐based whole genome sequencing (WGS) was performed using genomic DNA extracted from the blood of the two affected calves and both dams, and sperm of the common sire.

**Results:**

The cases were presented with a CP Veau II, permanent recumbency, strabismus, tachycardia, and tachypnea. Echocardiographic findings were consistent with tetralogy of Fallot associated with patent foramen ovale (pentalogy of Fallot). Necropsy examination identified hydrocephalus in addition to CP and confirmed the clinical diagnosis of pentalogy of Fallot. The calves were diagnosed with a complex syndromic form of CP with pentalogy of Fallot and hydrocephalus. Analysis of the breeding data showed that 19/45 recorded offspring of the sire were not viable. Genetic analysis identified a missense variant in *WDR33* that was heterozygous in both analyzed cases and in an estimated 40% of the paternal gametes of the mosaic founder, but absent in both dams and controls.

**Conclusions and Clinical Importance:**

This study alerts veterinarians and breeders to the potential occurrence of dominant de novo mutations in cattle and emphasizes that, in herds using a natural service sire, the consequences of an asymptomatic germline mosaic can be important.

**Abbreviations**
CBC: complete blood count
CP: cleft palate
gnomAD: genome aggregation database
OMIA: online mendelian inheritance in animals
NCBI: national center for biotechnology information
RI: reference intervals
WDR: WD repeat
WGS: whole‐genome sequencing

## 1 Introduction

Embryonic formation of the palate involves a complex sequence of processes that require precise coordination of cellular activities, including migration, growth, differentiation, and programmed cell death [1, 2]. Cleft palate (CP) is a congenital defect characterized by a fissure of the roof of the mouth produced by failure of the two maxillae to unite during embryonic development [3]. The Veau classification (1931) is one of the main systems used to categorize CP based on its morphologic features. It consists of four categories: Veau I, which refers to clefts of the soft palate; Veau II, which includes clefts involving both the soft and hard palates and extends to the incisive foramen; Veau III, which refers to clefts of the soft and hard palates extending unilaterally through the alveolus; and Veau IV, which describes bilateral clefts involving the soft and hard palates, extending bilaterally through the alveolus [4]. In humans, CP is one of the most common craniofacial congenital anomalies, with an estimated prevalence ranging from 1 to 7 in 1000 newborns [1]. Cleft palate can be classified into syndromic and non‐syndromic forms. The development of CP might have a genetic cause, with more than 200 human genes known to be involved, or be related to environmental factors (e.g., folate deficiency, maternal obesity). In human medicine, syndromic forms of CP account for approximately 27% of cases, whereas non‐syndromic forms, divided into isolated CP and CP with other abnormalities, account for 55% and 18%, respectively [5].

Both syndromic and non‐syndromic forms of CP with non‐hereditary and hereditary causes have been reported in cattle. Ingestion of teratogenic alkaloid‐containing plants between the 40th and 70th day of gestation (e.g., *Lupinus caudatis* *Lupinus sericeus* ) may lead to the development of “crooked calf disease,” in which affected animals exhibit a syndrome in which CP is associated with one or more musculoskeletal defects, such as arthrogryposis, torticollis and scoliosis [6]. In addition, it has been hypothesized that the ingestion of selenium‐rich plants (e.g., *Astragalus pectinatus,* *Astragalus racemosus* ) alone or in combination with manganese deficiency might lead to the development of a syndromic form of CP with musculoskeletal disorders (e.g., arthrogryposis, kyphoscoliosis) [7]. According to the Online Mendelian Inheritance in Animals (OMIA), monogenic recessive syndromic and non‐syndromic forms of CP have been reported in cattle [8]. The causative variant has been identified in three recessively inherited syndromic forms of CP. These are the *CHRNB1*‐ related arthrogryposis multiplex congenita in Red Danish (OMIA002022‐9913), *PIGH*‐related arthrogryposis lethal syndrome in Belgian Blue (OMIA001953‐9913), and *ACAN*‐related dwarfism in Dexter and Miniature Highland (OMIA 001271‐9913) [9, 10, 11]. In addition, a single recessively inherited *MYH3*‐related non‐syndromic form of CP has been described recently in Limousin (OMIA002590‐9913) [12, 13].

Two paternal half‐sibling crossbred calves with syndromic CP were identified. Our aim was to report the clinicopathologic phenotype of a complex syndromic form of CP observed in the progeny of a single Belgian Blue sire and identify the putative genetic cause using a multiple case trio‐based whole genome sequencing (WGS) approach.

## 2 Materials and Methods

### 2.1 Animals

Our study did not require regulatory or institutional ethical approval because it was not experimental, but rather part of clinical and pathological veterinary diagnostic evaluation. All animals in the study were examined with the consent of their owners and were treated according to ethical standards. The study included two affected calves (cases), their common sire, and two dams, and genomic data of 5576 cattle control genomes. Case 1 was a 2‐day‐old female and case 2 was a 4‐day‐old male. The farm of origin was a beef suckler herd of 50 cows with a single natural service sire on a spring seasonal system. The common sire of the calves was a Belgian Blue, and the dams were Charolais and Simmental (dams from case 1 and 2, respectively).

### 2.2 Clinical and Pathological Investigations

Two paternal half‐sibling Belgian Blue cross calves (cases 1 and 2) with CP were referred to the Clinic for Ruminants of the University of Bologna. The two referred calves were hospitalized at the clinic where they underwent a thorough physical examination accompanied by CBC and serum biochemistry. In addition, in case 2, an arterial blood gas analysis, a coagulation profile, and a transthoracic echocardiography were performed. The echocardiographic reference intervals (RI) were considered as previously reported [14].

Both animals were euthanized because of the poor prognosis and submitted for necropsy.

### 2.3 Genetic Analysis and DNA Extractions

Genomic DNA was obtained from the affected animals (EDTA blood samples), their respective dams (EDTA blood samples) and the common sire (semen sample) using the Promega Maxwell RSC DNA system (Promega, Dübendorf, Switzerland).

### 2.4 Whole Genome Sequencing and Variant Calling

A paternal half‐sibling trio‐based WGS approach using the Illumina NovaSeq6000 (Illumina Inc., San Diego, CA, USA) was performed on the genomic DNA extracted from EDTA preserved blood of the two affected calves, their two dams and from semen of the common sire. The sequenced reads were mapped to the ARS‐UCD1.2 reference genome, resulting in an average read depth of approximately 14.7× in the five sequenced genomes, and single‐nucleotide variants and small indel variants were called [15]. The applied software and steps to process fastq files into binary alignment map (BAM) and genomic variant call format files were in accordance with the 1000 Bull Genomes Project processing guidelines of run 7 [16], except for the trimming, which was performed using fastp [17]. Further preparation of the genomic data was performed as reported previously [18]. The effects of the above variants were functionally evaluated with snpeff v4.3 [19], using the National Center for Biotechnology Information (NCBI) Annotation Release 106 (https://www.ncbi.nlm.nih.gov/genome/annotation_euk/Bos_taurus/106/; acceded on 20 September 2024). This resulted in the final VCF file, comprising individual variants and their functional annotations. To identify private variants, we compared the genotypes of the cases with 1036 cattle genomes of different breeds sequenced as part of the ongoing Swiss Comparative Bovine Resequencing project. Integrative Genomics Viewer (IGV) [20] software version 2.0 was used for visual evaluation of genome regions containing potential candidate variants.

### 2.5 Variant Validation and Selection of Candidate Variants

The comprehensive variant catalogue of run 9 of the 1000 Bull Genomes Project was used to investigate the allele distribution of variants within a global control cohort allowing the exclusion of common variants [16]. The entire data set includes 5116 cattle genomes including 576 from the Swiss Comparative Bovine Resequencing project, from a variety of breeds (> 130 breeds indicated).

PolyPhen‐2 [21], SIFT [22] and SNAP [23] were used to predict the biological consequences of the detected missense variant. For cross‐species WDR33 protein sequence alignments, the following NCBI protein accessions were considered: NP_001193007.1 ( *Bos taurus* ), NP_060853.3 ( *Homo sapiens* ), XP_001139756.1 ( *Pan troglodytes* ), XP_028686443.1 ( *Macaca mulatta* ), XP_038281856.1 ( *Canis lupus* ), NP_083142.2 ( *Mus musculus* ), NP_001100868.2 ( *Rattus norvegicus* ), and XP_015132674.1 ( *Gallus gallus* ).

### 2.6 Sequence Accessions

All references to the bovine *WDR33* gene correspond to the NCBI accessions NC_037329.1 (chromosome 2, ARS‐UCD1.2), NM_001206078.1 (*WDR33* mRNA), and NP_001193007.1 (WDR33 protein). For the protein structure of WDR33, the Uniprot database [24] and InterPro [25] with accession number E1BCT7 were used.

## 3 Results

### 3.1 Clinical Phenotype

On physical examination, calf 1 presented with muscular weakness and left lateral recumbency. The calf was unable to assume a quadrupedal stance and, if passively positioned in sternal recumbency, was able to maintain the position for only a few minutes. The oral cavity showed a cleft palate affecting both the hard and soft palates and was classified as a Veau II form of CP. The suckling reflex was absent, whereas the cranial and spinal reflexes were present. The calf had tachycardia (150 heart beats/min) and tachypnea (44 respirations/min). The oculoconjunctival mucosal membranes were congested and the episcleral vessels were injected. In the right eye, a long and narrow opacification in the cornea was noticed. In addition, bilateral nystagmus and divergent strabismus of the left eye also were identified. The CBC was normal. On serum biochemistry, hypocholesterolemia (30 mg/dL; reference interval [RI]: 80–120), hyperphosphatemia (8.85 mg/dL; RI: 5.6–6.5) and hypochloremia (93.3 mEq/L; RI: 5.6–6.5) were identified.

On physical examination, calf 2 presented in right lateral recumbency with opisthotonos. If passively positioned in sternal recumbency, it was able to maintain the position for only a few minutes. Asymmetry of the splanchnocranium was noticed, with a marked protuberance on the left zygomatic bone. As in case 1, a Veau II form of CP was observed. Bilateral mucopurulent nasal discharge was present. The oculoconjunctival mucous membranes were pink, and divergent strabismus of the left eye was noted. The carotid pulse was clearly visible; the jugular veins appeared flat and empty, and pressure on the lower extremity of the vein failed to produce any degree of filling. The calf had tachycardia (185 heart beats/min) and tachypnea (61 respirations/min). A CBC disclosed moderate neutrophilia (9710/μL; RI: 600–4000) and lymphopenia (1400/μL; RI: 2500–7500). Echinocytes were observed on the blood smear. On serum biochemistry, marked hyperlactatemia (6.3 mmol/L; RI: 0.60–2.20), increased creatine kinase activity (637 U/L; RI: 105–409) and hypocholesterolemia (22 mg/dL; RI: 80–120) were noted. Coagulation parameters were within the normal range, and arterial blood gas analysis showed severely decreased oxygen partial pressure (42.5 mmHg; RI: > 90) and increased partial pressure of carbon dioxide (54 mmHg; RI: 35–44).

### 3.2 Echocardiographic Examination

**FIGURE 1:**
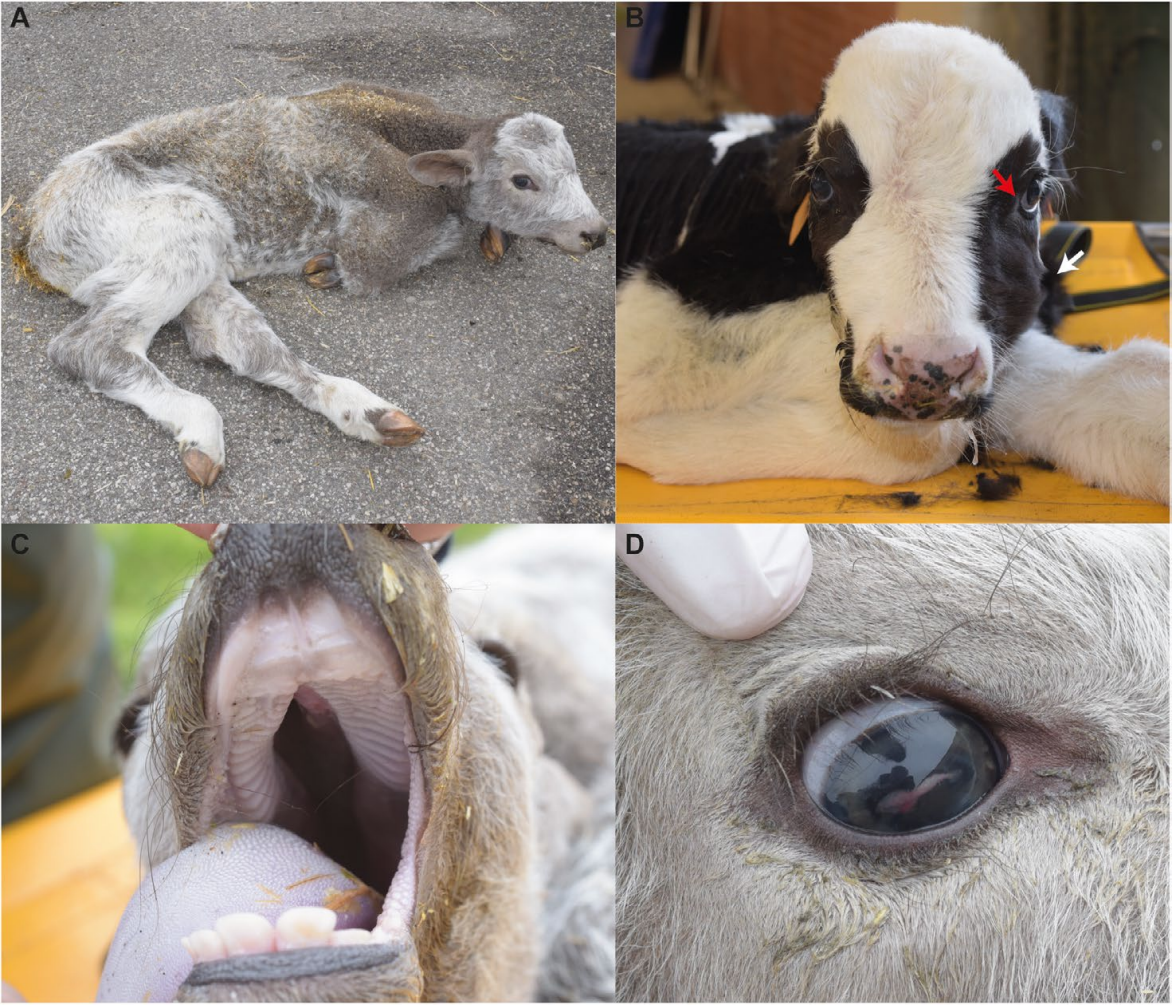
Complex syndromic form of cleft palate with pentalogy of Fallot and internal hydrocephalus in the progeny of a Belgian Blue sire. (A), Case 1 when positioned in sternal recumbency. Note the woolly and dull haircoat. (B) Case 2 when positioned in sternal recumbency. Note the asymmetry of the splanchnocranium with a marked protuberance on the left zygomatic bone (white arrow) and the divergent strabismus of the left eye. (C), Case 1 showing cleft palate affecting both the hard and soft palates (cleft palate Veau II form). (D) Case 1 particular of the right eye. Note long and narrow opacification in the cornea.

**FIGURE 2:**
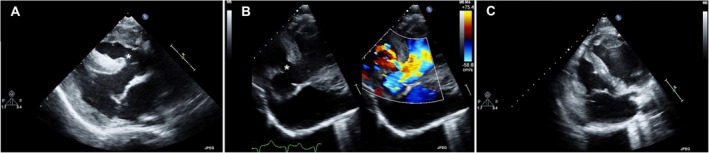
Echocardiographic findings in a crossbred calf with a complex syndromic form of cleft palate with pentalogy of Fallot and internal hydrocephalus. (A), Two‐dimensional image obtained from the right parasternal long‐axis view. Note the right ventricular wall thickening, the overriding aorta, and the ventricular septal defect (asterisk). (B), Two‐dimensional image obtained from a left parasternal apical view optimized to show the overriding aorta with and without concomitant color Doppler (right and left panels, respectively). Note the intracardiac left‐to‐right shunting flow moving through the ventricular septal defect (asterisk). (C), Two‐dimensional image obtained from a left parasternal apical view optimized to show the right‐sided cardiac chambers. Note the right atrial dilatation and the right ventricular free wall thickening.

Complete transthoracic echocardiography was performed only in case 2. Two‐dimensional images obtained from the right parasternal long‐axis view subjectively showed moderate thickening of the right ventricular walls (end‐diastolic thickness of the right ventricular free wall: 14 mm (Figure 1; Figure 2A). No right ventricular volume overload was documented (end‐diastolic and end‐systolic right ventricular internal diameter): 30 and 22 mm, respectively (RI: 33 ± 9 mm and 20 ± 5 mm, respectively), but subjectively severe right atrial dilatation was identified (66 mm; RI: 32 ± 7 mm). The inlet of cava veins and the coronary sinus were markedly distended. Similarly, distension of the caudal vena cava and hepatic veins was identified using a focus transdiaphragmatic view. Interestingly, a large great artery, expected to be the aorta, was identified overriding the interventricular septum with a large ventricular septal defect (diameter: 8 mm; Figure 2A). At that level, color flow Doppler documented a predominantly left‐to‐right flow (peak velocity: 3 m/s; pressure gradient: 36 mmHg). On inspection of the interatrial septum, two‐dimensional analysis allowed identification of a concomitant defect at the level of the anatomic region of the foramen ovale (diameter: 11 mm). On color Doppler interrogation, the defect was associated with a predominantly left‐to‐right shunt. In the right parasternal short‐axis view, the pulmonic valve leaflets appeared irregularly thickened and tethered, and systolic valve doming was appreciable. On color Doppler interrogation, antegrade flow acceleration was observed during systole, and mild valvular pulmonic insufficiency was noted during diastole. The peak velocity of these flows could not be precisely measured by continuous wave Doppler because the animal was uncooperative, and its particular chest conformation precluded optimal cursor alignment. The remaining echocardiographic parameters, including left ventricular end‐diastolic and end‐systolic internal diameters (51 and 37 mm, respectively; RI: 54 ± 11 mm and 35 ± 9 mm, respectively), left ventricular systolic function (fractional shortening: 28%; RI: 35% ± 10%) and left atrial dimensions (left atrial‐to‐aortic root ratio: 1.2) were judged to be normal. The aforesaid abnormalities, including the remodeling of the right cardiac chambers, the overriding aorta and the ventricular septal defect associated with predominantly left‐to‐right flow, were clearly evident even from the echocardiographic views obtained in left recumbency (Figure 2B,C). The findings were most consistent with a provisional diagnosis of tetralogy of Fallot associated with concurrent patent foramen ovale (also termed pentalogy of Fallot) [26].

### 3.3 Pathological Phenotype

**FIGURE 3:**
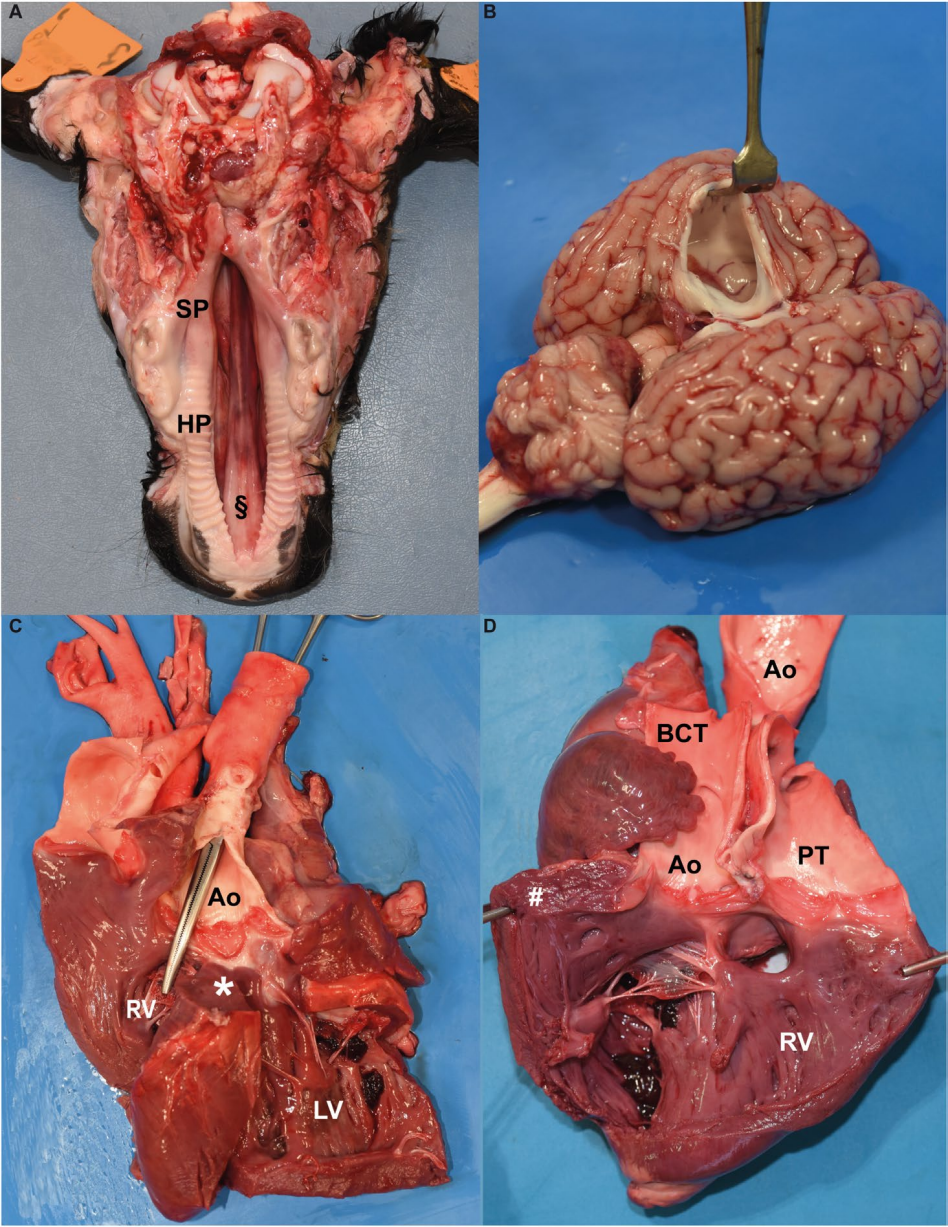
Gross pathological findings in crossbred calves with a complex syndromic form of cleft palate with pentalogy of Fallot and internal hydrocephalus. (A), Case 1, cleft palate Veau II form. Cleft affecting both the hard palate (HP) and soft palate (SP). The nasal septum (§) is also visible. (B) Case 2, Hydrocephalus. A section through the left cerebral cortex has been made to visualize the dilation of the left lateral ventricle. (C) Case 2, heart. View after opening of the right (RV) and left (LV) ventricles and removal of the dorsal part of the interventricular septum. Note the overriding aorta (Ao) above the interventricular septal defect (*). (D) Case 1, heart. View after opening of the right ventricle (RV). Note the marked hypertrophy of the RV (#) and the origin of both the aorta (Ao) and the pulmonary trunk (PT) from the right ventricle. BCT, brachiocephalic trunk.

Besides the CP (Figure 3A), necropsy in both cases identified internal hydrocephalus (Figure 3B) and a complex heart malformation. An in‐depth dissection of the heart showed ventricular septal defect, overriding aorta, pulmonary stenosis, right ventricular hypertrophy, and patent foramen ovale, confirming the tentative echocardiographic diagnosis of pentalogy of Fallot (Figure 3C,D).

Calf 1 also had aspiration bronchopneumonia involving the cranioventral lobes of the lung, especially the apical left lobe. The haircoat was woolly, dull, and thin.

Additionally, calf 2 had an extra pair of ribs (14 instead of 13) and a malformation of the torus linguae, which was bilobed. The apex of the tongue also was abnormal, being deviated laterally to the left side. Additional findings were areas of hyperemia and hyperkeratosis in the ruminal and reticular mucosa, abomasal subserosal hemorrhage, isolated simple cysts in the left kidney, and partial ectopia of the spiral loop of the ascending colon.

The clinical, echocardiographic, and pathological examinations characterized the two calves' condition as a syndromic form of CP accompanied by pentalogy of Fallot and internal hydrocephalus.

### 3.4 Genetic Analysis

Analysis of breeding data showed that the Belgian Blue bull produced a total of 45 calves from dams of different breeds during the season in which the two affected calves were born, of which 19 (including the two calves referred) died or were euthanized within the first week of life. The local practitioner carried out on‐site necropsy examinations on three calves and observed CP in all, cardiac malformations in two, hydrocephalus in one (one not tested), and hindlimb hypoplasia (right hindlimb agenesis and rudimentary femur in the left hindlimb) in one. Unfortunately, these calves were not sampled for genetic evaluation.

Given that a total of 19 of 45 offspring of the sire died unexpectedly and without any other evident reason during the first week of life, and that five of them were most likely affected by the same syndrome (cases 1 and 2), we speculate that the remaining 14 might have been affected by the same anomaly. However, these 14 remaining animals did not undergo necropsy, and therefore the cause of death could not be determined. Based on the case history and pedigree, we hypothesized that the current cases could be explained by a dominant de novo mutation. Therefore, we hypothesized that the sire could be an asymptomatic gonadal mosaic.

**FIGURE 4:**
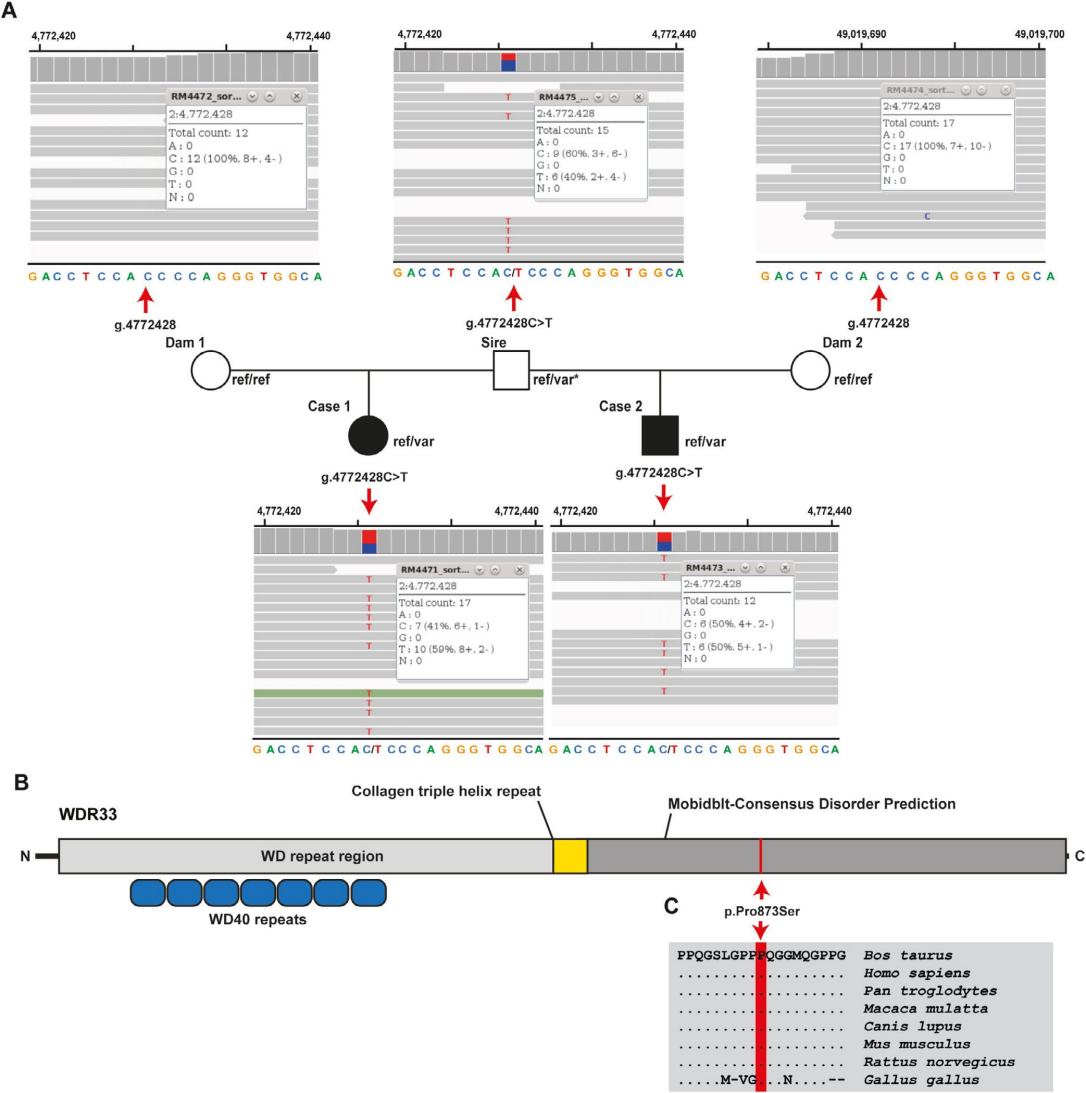
Heterozygous *WDR33* missense variants in two paternal halfsiblings crossbred calves affected by a complex syndromic form of cleft palate with pentalogy of Fallot and internal hydrocephalus. (A), Pedigree of the two cases IGV screenshot presenting the Chr2: G.4772428C>T variant heterozygous in the affected calves and semen of the sire (*) and homozygous wildtype in both dams revealed by whole‐genome sequencing. (B), Schematic representation of the bovine WDR33 protein and its functional domains. The red line indicates the position of the variant in the protein. (C), Multiple sequence alignment of WDR33 protein encompassing the region of the p. variant demonstrates complete evolutionary conservation across species.

Variant data analysis confirmed the suspected parentage. Filtering of WGS for private shared heterozygous variants, present in the sequenced genomes of cases 1 and 2 excluding the sire and absent in 1036 available control genomes (including the two dams), identified 24 protein‐coding variants common to both cases (Table 1, Table S1). Analysis of the occurrence of these variants in the global control cohort of genomes from a variety of breeds [16] determined that, from the identified protein‐changing heterozygous variants with a predicted moderate impact, only 6 were privately present in the genomes of both sequenced cases. Of the variants that remained, only one in the *WD Repeat Domain 33* (*WDR33*) gene was predicted to have an effect on a functional candidate gene for the phenotype under study. It was a heterozygous missense variant at Chr2:4772428C>T in exon 16 of *WDR33* (NM_001206078.1: c.2617C>T). It is predicted to exchange the encoded amino acid of WDR33 at position 873 (NP_001193007.1: p.Pro873Ser; Figure 4B). Furthermore, the proline‐to‐serine substitution affects a highly conserved residue (Figure 4C) and was predicted to be deleterious by three different tools (PolyPhen‐2 score, 41% deleterious; SIFT score, 43% deleterious; SNAP score, 56% deleterious; Table S2).

**TABLE 1:** Results of variant filtering of the affected calves using the whole‐genome sequence data.

Filtering step	Homozygous variants	Heterozygous variants
All variants in case 1	2 601 986	4 875 617
All variants in case 2	2 615 697	5 165 773
Private variants in cases 1 and 2	0	4638
Private variants in cases 1 and 2 absent in the dams and in a global cohort of 5576 cattle control genomes	0	589
Protein‐changing private variants absent in the dams and in a global cohort of 5576 cattle control genomes	0	6

This variant was found to be heterozygous in the blood DNA of both cases and the sperm DNA of the common sire but was absent from the blood DNA of both dams (Figure 4A). The variant allele was present in heterozygous form in the sire and in both affected calves at different ratios. This finding indicates that the bull might carry the variant allele at a lower level than the reference allele, with an estimated 40% compared to approximately 50% in both cases (Figure 4A). Therefore, we suggest that the sire was a founder mosaic, at least gonadal, and possibly also somatic. However, the latter could not be confirmed because of a lack of appropriate samples.

The genetic findings were most consistent with a likely pathogenic dominant de novo mutation in *WDR33* as the underlying cause of the observed congenital syndrome.

## 4 Discussion

In our study, thorough clinical, pathological, and genetic evaluation of two crossbred paternal half‐sibling calves with a complex syndromic form of CP with pentalogy of Fallot and internal hydrocephalus was performed. We present evidence for the occurrence of a dominant de novo missense mutation in *WDR33* as a likely pathogenic variant for a syndromic bovine form of CP. The paternal half‐sibling trio‐based WGS approach identified six protein‐changing variants that were simultaneously exclusively heterozygous in the genome of the affected calves, excluding the sire. After gene function analysis, taking into account the occurrence of the variant allele in a global control cohort, the rarity of the variant, and *in silico* effect predictions, the identified heterozygous missense variant in *WDR33* was determined to be the most probable genetic cause of the observed congenital condition. Large data from human genome sequencing studies presented in the Genome Aggregation Database (gnomAD) showed that the probability of loss‐of‐function intolerance score for *WDR33* was 1, meaning that this gene falls into the class of loss‐of‐function haploinsufficient genes [27]. In addition, according to gnomAD, the *WDR33* Z‐score for missense and synonymous variants is relatively high (5.61), meaning that this gene tends to fall into the class of intolerance to variation in terms of missense and synonymous variants [28]. Considering these, we hypothesize that the identified variant in *WDR33* might disrupt the formation of heterotrimeric or multiprotein complexes of the encoded protein. Our findings suggest that the common sire of the analyzed cases was an asymptomatic gonadal mosaic of a de novo mutation. Our study provides an example of paternal germline mosaicism in cattle as previously reported for achondrogenesis type II *COL2A1*‐related (OMIA001926‐9913) [29, 30], chondrodysplasia *FGFR3*‐related (OMIA001703‐9913) [31] and osteogenesis imperfecta *COL1A1*‐related (OMIA002127‐9913) [32]. To accurately estimate the exact percentage of paternal germline mosaicism, a more quantitative method such as droplet digital PCR could have been used, as previously reported [32]. Unfortunately, DNA samples from the sire were not available because they were used for WGS.

*WDR33* encodes the pre‐mRNA 3′ end processing WDR33 protein that is a member of the WD repeat (WDR) protein family, which plays a pivotal role in several essential biological functions such as signal transduction, transcription regulation, and apoptosis [33]. Variants affecting WDR proteins have been associated with a large spectrum of disorders in humans such as ciliopathies, as well as immunological, ophthalmological, skeletal, neurological, and endocrine disorders [34]. Currently, the involvement of *WDR33* in diseases is poorly understood. According to the MalaCards Human Disease database, *WDR33* is associated with the dominantly inherited atrial septal defect [35, 36], a condition characterized by an atrial septal defect with variable clinical expression [37]. In the presented bovine cases, given the potential involvement of the gene in congenital heart defects, it is plausible that it could be involved in more severe conditions such as the observed pentalogy of Fallot, broadening the spectrum of possible associated congenital heart defects. In addition, *WDR33* has been associated with neurodevelopmental disorders in human patients, and *Wdr33* heterozygous mutant mice exhibit abnormal prepulse inhibition implicating a role for *WDR33* in neurodevelopment [38, 39, 40]. In fact, the presence of a neurological disorder with presumed neurolocalization to the prosencephalon and brainstem, confirmed by pathology where hydrocephalus was diagnosed, also was observed in the calves presented in our study. Moreover, *WDR33* has interactions with 156 different genes [41]. Among these various interacting genes, four (*RAF1*, *BRD4*, *ATRX*, and *TKT*) have been associated with syndromic congenital conditions involving craniofacial abnormalities, heart defects, and neurological disorders. Specifically, pathogenic heterozygous variants in *RAF1* cause LEOPARD syndrome 2 and Noonan syndrome 5 in humans (OMIM 164760), where affected patients have, among other features, short stature, dolichocephaly, hypertrophic cardiomyopathy, pulmonary valve stenosis, and neurodevelopmental delay [42, 43]. In human patients, heterozygous variants in *BRD4* can cause Cornelia de Lange syndrome 6 (OMIM 620568) characterized by microcephaly and long philtrum, CP, distal limb defects, retarded growth, heart defects, and neurodevelopmental delay [44, 45, 46]. In addition, *ATRX* is associated with a dominantly inherited form of alpha‐thalassemia/impaired intellectual development syndrome in human patients (OMIM 301040). Patients with this syndrome, among other features, have retarded growth, microcephaly, ventricular septal defects, limb malformations, neurodevelopmental delays, and cerebral atrophy [47, 48]. Finally, recessively inherited pathogenic alleles in *TKT* have been reported to be associated with short stature, developmental delays, and congenital heart defects (OMIM 617044) [49]. In particular, patients with this syndrome have complex heart defects including ventricular septal defects, atrial septal defect, patent foramen ovale, and patent ductus arteriosus, and some patients also may have cataracts [49]. Interestingly, in our study both calves presented with craniofacial abnormalities, heart defects, and a neurological condition, and one of the reported cases had an opacification in the cornea compatible with a congenital cataract.

Most of the clinically identified disease‐causing variants in WDR are located on the surface of the protein and are thought to disrupt its binding to other proteins [33, 34, 50, 51]. Based on our results, we speculate that the observed phenotype in the calves described in our study might be caused by an impaired interaction between *WDR33* and the aforementioned interacting genes. However, it cannot be excluded that the phenotype exhibited by affected calves is solely caused by dysfunction of the mutant WDR33 protein. Additional experimental studies would be required to functionally validate the postulated causative role of the candidate variant in the observed syndromic condition. Therefore, future studies should be aimed at investigating the role of pathogenic *WDR33* variants and their role in the development of syndromes involving craniofacial anomalies, heart defects, and neurological disorders.

Herein, we have identified a novel phenotype of a complex syndromic form of CP in cattle, including pentalogy of Fallot and hydrocephaly, associated with a dominantly inherited likely pathogenic de novo mutation in the bovine *WDR33* gene. We propose that this represents the first *WDR33*‐related form of a congenital syndrome in a mammalian species, adding this relatively uncharacterized gene to the list of candidates for similar disorders in animals and humans. Our results might serve as a starting point for further research into the function and mechanisms of *WDR33* in the context of syndromic forms of CP, congenital heart defects, and neurodevelopmental disorders. Our study emphasizes that the genetics of inherited disorders in well‐phenotyped large animals, such as cattle, is a valuable model system for studying fundamental aspects of gene function. Our study also alerts veterinarians and cattle breeders to the potential emergence of dominantly inherited paternal germline mutations, a relatively common mechanism in the origin of genetic disorders also occurring in cattle, and emphasizes that in herds using a natural service, if one sire is affected, the consequences can be clinically and economically important.

## Disclosure

Authors declare no off‐label use of antimicrobials.

## Ethics Statement

Authors declare no institutional animal care and use committee or other approval was needed. Authors declare human ethics approval was not needed for this study.

## Conflicts of Interest

The authors declare no conflicts of interest.

## Supporting information


**Table S1.** List of the remaining variants after the comparison to the global control cohort of genomes of different breeds and after IGV visual inspections, revealing 6 protein‐changing private variants with a predicted moderate impact only present in the affected calves excluding the sire.

**Table S2.** Pathogenicity prediction results for the 6 heterozygous protein‐changing variants exclusively present in the genome of the affected calves excluding the sire and absent in the global control cohort of genomes of a variety of breeds.
