# Supplementary material for: De Novo Missense Variant in Bovine WDR33 Associated With a Complex Syndromic Form of Cleft Palate With Pentalogy of Fallot and Internal Hydrocephalus
Source: J Vet Intern Med. 2025 Jun 17;39(4):e70144. doi: 10.1111/jvim.70144 (PMC12171993; doi:10.1111/jvim.70144)
Supplement: Supplementary file 2 — Table S2. Pathogenicity prediction results for the 6 heterozygous protein‐changing variants exclusively present in the genome of the affected calves excluding the sire and absent in the global control cohort of genomes of a variety of breeds. [file JVIM-39-e70144-s002.pdf]

Supplementary Table S2. Pathogenicity prediction results for the 6 heterozygous protein-changing variants exclusively present in the genome of the affected calves excluding the sire and absent in the global control cohort of 5576 genomes of a variety of breeds.

**Gene**	**OMIM**	**Associated disorder/ gene function**	**Protein change**	**Predicted effect[1]**
WDR33	618082	Subunit of cleavage and polyadenylation specificity factor (CPSF), which mediates 3-prime cleavage and AAUAAA-dependent polyadenylation of mRNAs	p.Pro873Ser	Deleterious
RP1L1	608581	Occult macular dystrophy; Retinitis pigmentosa 88	p.Val1344Ile	Neutral
C15H11orf53	NA	Uncharacterized	p.Gly151Arg	Neutral
GRIK5	600283	Cation-permeable ligand-gated ion channel, gated by L-glutamate and the glutamatergic agonist kainic acid	p.Ala425Thr	Neutral
TSKS	608253	Downregulated in cancerous testicular tissue compared with adjacent normal tissue	p.Pro123Ser	Neutral
MGC137036	NA	Uncharacterized	p.Val49Met	Neutral

OMIM, Online Catalog of Human Genes and Genetic Disorders; [1] based on PolyPhen-2 [22], SIFT [23] and SNAP [24] results; NA, not available.
